# Baicalein prevents 6-OHDA/ascorbic acid-induced calcium-dependent dopaminergic neuronal cell death

**DOI:** 10.1038/s41598-017-07142-7

**Published:** 2017-08-21

**Authors:** Sheng-Fang Wang, Liang-Feng Liu, Ming-Yue Wu, Cui-Zan Cai, Huanxing Su, Jieqiong Tan, Jia-Hong Lu, Min Li

**Affiliations:** 1 State Key Laboratory of Quality Research in Chinese Medicine, Institute of Chinese Medical Sciences, University of Macau, Taipa Macao SAR, China; 2 School of Chinese Medicine, Hong Kong Baptist University, Kowloon Tong, Kowloon Hong Kong; 3 Mr. and Mrs. Ko Chi Ming Centre for Parkinson’s Disease Research, Hong Kong Baptist University, Kowloon, Hong Kong; 4 Limin Pharmaceutical Factory, Livzon Pharmaceutical Group Inc. Shaoguan, Guangdong, China; 5 State Key Laboratory of Medical Genetics, Xiangya Medical School, Central South University, Changsha, Hunan China

## Abstract

6-OHDA plus ascorbic acid (AA) has long been used to induce Parkinson’s disease in rodents, while only 6-OHDA is commonly used to induce cell damage in cellular PD models. AA was believed to act as an anti-oxidant to prevent the degradation of 6-OHDA; however, some studies suggested that AA dramatically enhanced the selectivity and toxicity of 6-OHDA. To understand the mechanisms by which 6-OHDA/AA induces cell death, we established a 6-OHDA/AA cell toxicity model in human dopaminergic neuroblastoma SH-SY5Y cells. We confirmed that the toxicity of 6-OHDA was dramatically increased in the presence of AA, and the toxicity can be prevented by a flavonoid, baicalein. Mechanistically, our research reveals that 6-OHDA/AA induces cell death mainly through the interruption of intracellular calcium homeostasis, which leads to calpain activation and mitochondrial damage. Baicalein prevents 6-OHDA/AA-induced intracellular calcium elevation as well as consequent mitochondria damage. Taken together, our study confirms that 6-OHDA/AA is a more sensitive model for inducing neuronal lesion *in vitro* and reveals the central role of intracellular calcium in 6-OHDA/AA-induced cell death. Our studies further show that baicalein prevents 6-OHDA/AA-induced cell death by inhibiting intracellular calcium elevation.

## Introduction

Parkinson’s disease (PD), a progressive neurodegenerative disease strongly associated with aging, is characterized by the selective death of dopaminergic neurons in *substania nigra* and the accumulation of cytoplasmic inclusions called Lewy bodies^1^. 6-OHDA is a classic PD toxin which induces neurotoxicity through causing the production of hydrogen peroxide and hydroxyl radicals, reducing GSH content and inhibiting SOD activity^2–5^. It was commonly believed that, in 6-OHDA models both *in vivo* and *in vitro*, AA is an antioxidant that prevents oxidation of 6-OHDA^6^. Moreover, it has been revealed that 6-OHDA/AA combination enhanced selectivity and toxicity of 6-OHDA compared with 6-OHDA treatment alone. However, the specific mechanisms of the action of 6-OHDA when it is used alone and when is it used together with AA are rarely investigated. Baicalein (BC) is one of the most effective antioxidants among the major flavonoids that are extracted from the root of Scutellaria baicalensis **(**Huangqin in Chinese); it has been reported to have anti-inflammatory and anti-oxidant properties^7, 8^. Recent studies have shown that baicalein protected against ß-amyloid peptide-(25–35) and 6-OHDA-induced neurotoxicity and neuronal injury secondary to ischemia insult *in vivo* and *in vitro*; whereas the mechanisms of neuroprotection is still unclear^9–11^. Here, we report the establishment of a cellular PD model of 6-OHDA/AA lesion on SH-SY5Y cells and demonstrate the mechanisms by which baicalein counteracts 6-OHDA/AA induced-calcium elevation and consequent cells death.

## Results

### Establishment of 6-OHDA/AA cell toxicity model and neuroprotective effects of baicalein on 6-OHDA/AA-induced cell death

**Figure 1:**
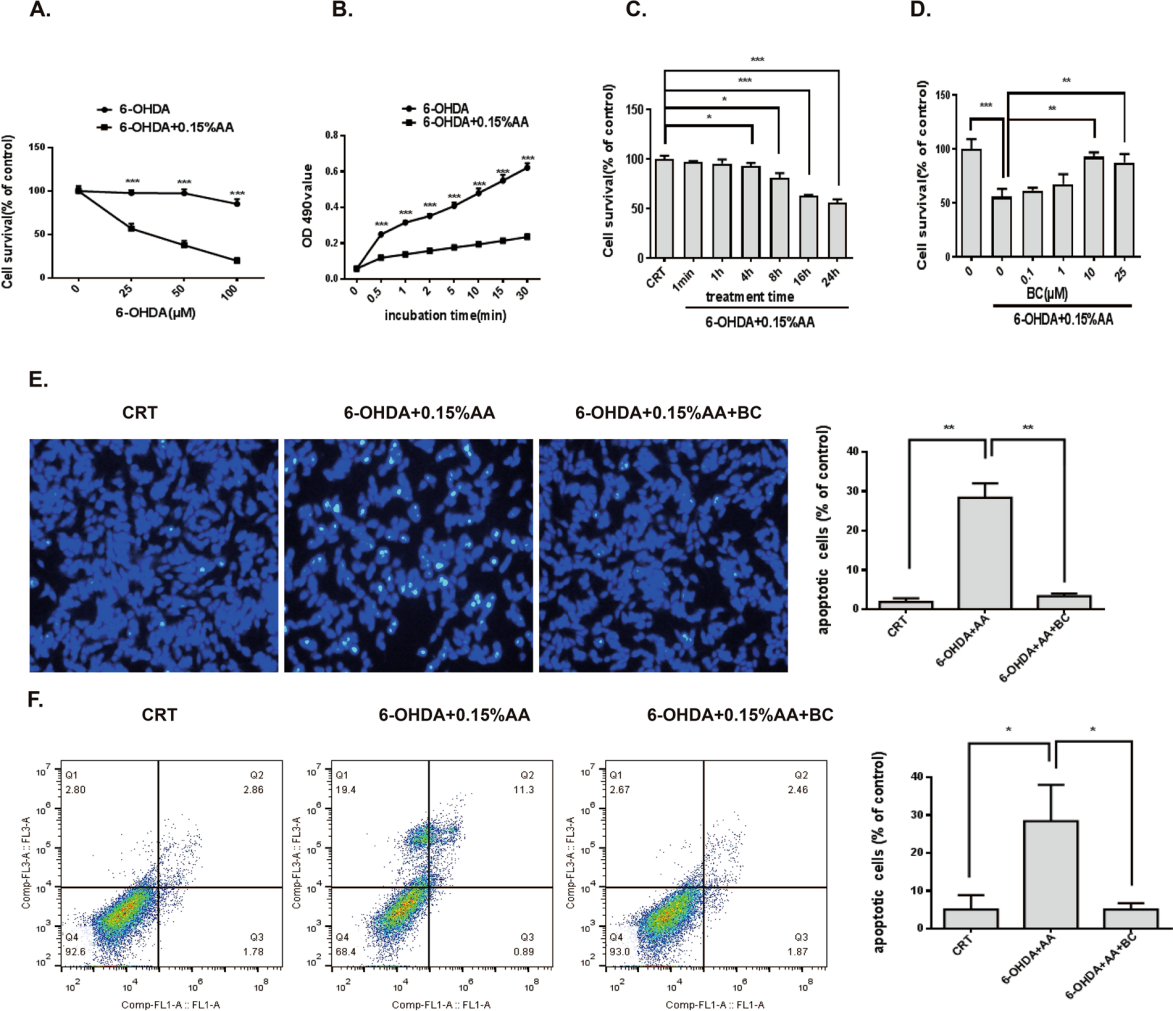
Neuroprotective effect of Baicalein against 6-OHDA/AA induced neurotoxicity on SH-SY5Y cells. (**A**) SH-SY5Y cells were treated with different concentrations of 6-OHDA alone or 6-OHDA/AA for 15 min, and then drug-containing medium was replaced with normal cell culture medium, and cells were incubated for 24 h. Cell viability was assessed by MTT assay. (**B**) 6-OHDA autodegradation was measured during the incubation with 0.15% AA and PBS for different time. The autodegradation rate was measured at 570 nm absorbance. (**C**) SH-SY5Y cells were treated with 25 μM 6-OHDA/AA for 15 min and incubated in normal culture medium for different time. (**D**) SH-SY5Y cells were treated with 25 μM 6-OHDA/AA in the presence or absence of 25 μM baicalein for 15 min and incubated in normal culture medium for 24 h. (**E**) SH-SY5Y cells were treated with 25 μM 6-OHDA/AA in the presence or absence of 25 μM baicalein for 15 min and incubated in normal culture medium for 24 h. DAPI staining was investigated using a fluorescence microscope InCell 2000 system. (**F**) Baicalein inhibited 6-OHDA/AA-induced cell apoptosis. SH-SY5Y cells were treated with 25 μM 6-OHDA/AA in the presence or absence of 25 μM baicalein for 15 min and incubated in normal culture medium for 6 h. Apoptotic cells were double-stained with Annexin V-PI and analyzed using flow cytometry. The results are the mean ± SD values obtained in three independent experiments (*p < 0.05, **p < 0.01, ***p < 0.001).

SH-SY5Y cells were treated with different concentrations of 6-OHDA (25 μM-100 μM) with or without 0.15% AA for 15 min and then incubated with normal cell culture medium for different times (1 min-24 h). Cell viability was determined using MTT assay. The data showed that exposure of the cells to different concentrations of 6-OHDA alone did not change the cell viability markedly till 100 μM (Fig. 1A), and cell viability remained at 95 ± 6% compared with control. Whereas when cells were exposed to 6-OHDA/AA, cell viability dramatically reduced in a dose-dependent and time-dependent manner (Fig. 1A and C), and 25 μM 6-OHDA/AA reduced cell viability to 55% ± 7% when compared with control. The data indicates that 6-OHDA and AA lead to synergistic cytotoxicity which cause higher cell death rate than 6-OHDA treatment alone. We also confirmed that the autodegradation rate of 6-OHDA maintained in 0.15% AA was much slower than that in the PBS (Fig. 1B), indicating the prevention of 6-OHDA degradation by AA.

Through a drug screening process, we found that the natural flavonoid baicalein exerted a nice protective effect on the 6-OHDA/AA model. As shown in Fig. 1D, bacalein dose-dependently prevented the toxicity of 6-OHDA/AA, increasing the cell viability from 55% ± 7% (6-OHDA/AA) to 87 ± 8% (6-OHDA/AA + 25 μM baicalein). These results indicated that bacalein counteracted 6-OHDA/AA-induced neurotoxicity in SH-SY5Y cells.

Cells suffering from apoptosis have a morphology characterized by nuclear fragmentation and nuclear condense, which can be determined by DAPI staining. After designed treatment as indicated, the samples were subjected to DAPI staining. As shown in Fig. 1E, DAPI nuclear staining was detected by a fluorescent microscope. Compared with the control group, the 6-OHDA/AA treatment group showed significantly increased nuclear condense rate. However, baicalein reversed 6-OHDA/AA-induced nuclear condense. To further confirm the preventive effects of baicalein against 6-OHDA/AA-induced cell apoptosis, SH-SY5Y cells were stained with apoptosis marker Annexin V/PI and analyzed with flow cytometry (Fig. 1F). The result indicated that baicalein rescued SH-SY5Y cells from 6-OHDA/AA-induced apoptosis.

### Prevention of 6-OHDA/AA-induced intracellular calcium elevation by baicalein

**Figure 2:**
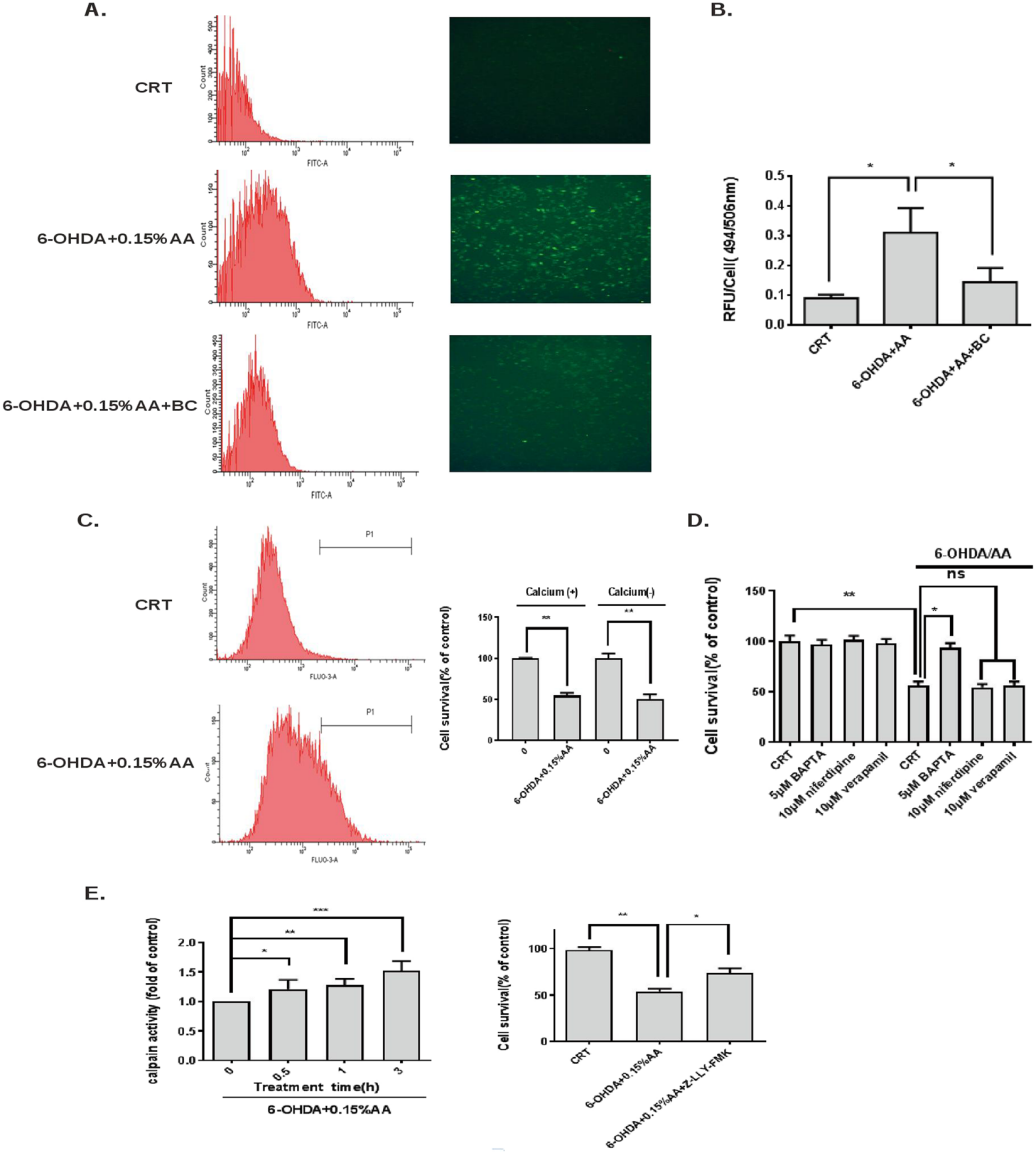
Neuroprotective effects of baicalein against 6-OHDA/AA-induced calcium activation in SH-SY5Y cells. (**A,B**) Baicalein inhibited 6-OHDA/AA-induced calcium elevation. SH-SY5Y cells were treated with 25 μM 6-OHDA/AA in the presence or absence of 25 μM baicalein and incubated for 0.5 h. Fluorescence intensity of the calcium was detected by flow cytometry (A, left panel) and confocal microscopy (A, right panel). The fluorescence signal was measured by a microplate fluorescence reader and expressed as relative fluorescence unit (RFU) per cells. (**B**). (**C**) Calcium efflux from cellular organelle mediated 6-OHDA/AA-induced cytotoxicity. The cells received the treatment as described in Figs 2A and B, in a calcium-free medium. The fluorescence intensity was measured by flow cytometry and the cell viability was measured by MTT assay. (**D**) Effect of calcium chelator and calcium channel blockers on 6-OHDA/AA-induced cell death. SH-SY5Y cells were treated with 25 μM 6-OHDA/AA, in the presence of 5 μM BAPTA, 10 μM Niferdipine or 10 μM verapamil and incubated for 24 h, the cell survival was measured by MTT assay. (**E**) SH-SY5Y cells were treated as described in the panel for 6 h, and the calpain activity was measured by a commercialized calpain activity kit using fluorometer reader (Left panel). The protective effect of calpain inhibitor was measured by MTT assay (right panel). The results are the mean ± SD values obtained in three independent experiments (*p < 0.05, **p < 0.01, ***p < 0.001).

The calcium signal, which has been reported to be required for caspase activation and calcium-dependent cysteine protease like calpain activation in the neuronal death, can be originate from extracellular and or intracellular calcium pool^12, 13^. In order to determine whether calcium-calpain activation is involved in 6-OHDA/AA-induced cell apoptosis in SH-SY5Y cells, the cells were treated as indicated in Fig. 2. 6-OHDA/AA treatment led to a significant elevation of calcium flux, whereas baicalein rescued cells via the inhibition of calcium release (Fig. 2A and B). To determine the source of the increased cytosolic calcium induced by 6-OHDA/AA, we checked the effect of 6-OHDA/AA on intracelluar calcium level in a calcium-free medium and found that 6-OHDA/AA still induced the increase of intracelluar calcium. This finding suggested that the source of increased cytosolic calcium is the intracellular calcium pool rather than extracelluar calcium influx (Fig. 2C). To further confirm the critical role of intracelluar calcium elevation in 6-OHDA/AA-induced toxicity, the cells were co-treated with either cell-permeable calcium chelator BAPTA (5 μM), or the specific calcium channel inhibitors Niferdipine (10 μM) and verapamil (10 μM). Consistently, BAPTA, but not Niferdipine nor Verapamil, rescued almost all (94 ± 4%) cells from 6-OHDA/AA-induced toxicity (Fig. 2D). We applied the same treatments to SH-SY5Y cells and measured calpain activity. The calpain activity was time-dependently activated after exposure to 6-OHDA/AA. When co-treated with calpain inhibitor Z-LLY-FM, up to 75 ± 5% cells could be rescued, indicating that calpain activation is involved in the cell toxicity induced by 6-OHDA/AA.

### Protective effect of baicalein on 6-OHDA/AA-induced mitochondrial damage

**Figure 3:**
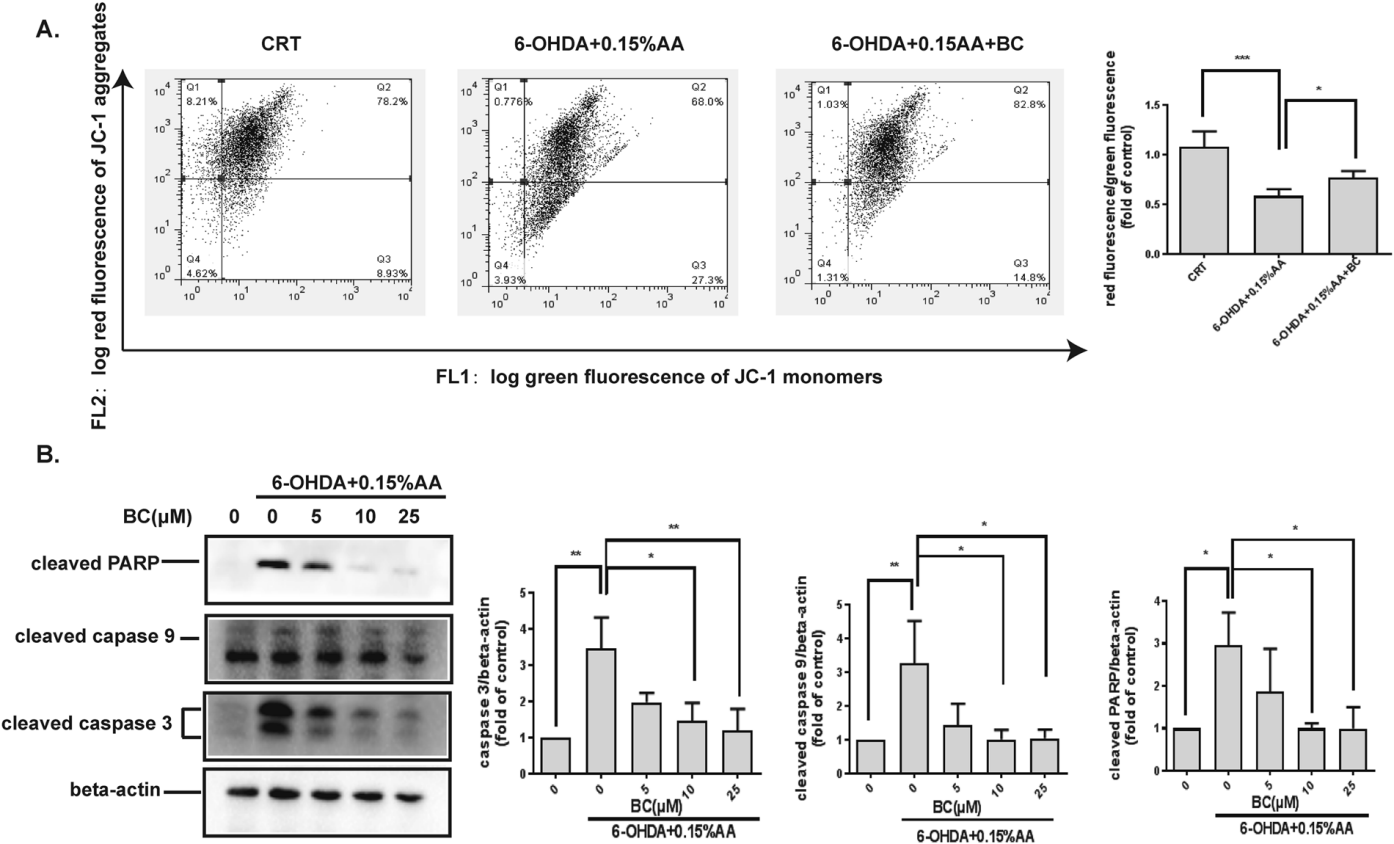
Neuroprotective effect of baicalein against 6-OHDA/AA-induced mitochondrial damage in SH-SY5Y cells. (**A**) Baicalein  attenuated 6-OHDA/AA-induced decrease of MMP. SH-SY5Y cells were treated as described. Samples were incubated with JC-1 dye, and red fluorescence and green fluorescence were measured. The ratio of red fluorescence to green fluorescence of the control was defined as 100%. Data shown are mean ± SD (n = 3). (**B**) Inhibition of baicalein on 6-OHDA/AA-induced activation of cleaved caspase3, cleaved caspase9 and cleaved PARP. Equal amounts of protein from total fraction of cells that have been treated with 6-OHDA/AA and different concentrations of baicalein for 12 h were analyzed via immunoblotting. The control was defined as 100%. The results are the mean ± SD values obtained in three independent experiments (*p < 0.05, **p < 0.01, ***p < 0.001).

It has been reported that 6-OHDA-induced neurotoxicity leads to mitochondrial dysfunction with a loss of mitochondrial membrane potential (MMP), which is a critical event in dopaminergic neuron degeneration^14^. To examine whether 6-OHDA/AA induces change in mitochondrial membrane potential, cells were treated as indicated in Fig. 3, and then the samples were incubated with JC-1 dye to monitor the MMP. The samples were analyzed by detecting the red fluorescence and green fluorescence ratio. Exposure of SH-SY5Y cells to 6-OHDA/AA decreased the ratio of red fluorescence/green fluorescence by about 55 ± 6% compared with control, which meant that 6-OHDA/AA induced a reduction in MMP. Co-treatment with baicalein attenuated this decrease (Fig. 3A).

The caspase family such as caspase 3 and caspase 9 have been implicated in cell apoptosis promoted by different death stimuli^15^. To demonstrate the apoptotic neuronal cell death induced by 6-OHDA/AA, cells were treated as indicated and then the protein expression levels of cleaved caspase3, cleaved caspaes9, and cleaved PARP were measured using immunoblotting analysis (Fig. 3B). Compared to the control group, 6-OHDA/AA treatment dramatically increased the apoptotic markers: cleaved caspase3, caspase9 and PARP, whereas baicalein significantly inhibited the activation of apoptotic markers in a dose-dependent manner.

## Discussion

6-OHDA model has been utilized for many years as a classic experimental model of PD. AA is a routine additive, used as an antioxidant to inhibit oxidation of 6-OHDA. Numerous reports on the mechanisms of 6-OHDA-inudced neuron cell death in *substantia nigra* striatum indicated that 6-OHDA caused cell death primarily by inducing progressive loss of dopaminergic neurons and incurring oxidative stress. Toxicity of 6-OHDA on neuroblastoma has been revealed to be increased in the presence of AA, which suggests that the 6-OHDA/AA-induced cellular lesion model should be regarded as a more specific PD model for further research. However, little is known about the molecular mechanisms involved in 6-OHDA/AA activity. In this work, we established a sensitive PD model using 6-OHDA/AA on SH-SY5Y cells and investigated the molecular mechanisms involved in calcium-calpain activation and mitochondrial dysfunction, including the decrease of MMP and the activation of the caspase family.

Through the novel 6-OHDA/AA toxicity model, our research sheds a new light on the mechanisms of 6-OHDA/AA-induced neurotoxicity. In our model, we observed a clearly increased intracellular calcium flux. It has long been known that calcium modulates a host of cell functions essential for cell survival disrupting the calcium homeostasis can initiate a cascade of pathological changes, including a massive activation of proteases and phospholipases which result in cytotoxicity to induce cell death. Several lines of evidence support the involvement of calcium in many neurotoxin-induced models^16, 17^. In our cellular model, cells were treated with low concentration of 6-OHDA/AA for very short period (15 min), and the cells undergo serious apoptosis, accompanied by intracellular calcium elevation and activation of calpain activity. The calcium chelating agent BAPTA as well as calpain inhibitor significantly attenuated 6-OHDA/AA-induced cell death. Calpain has been reported to participate in necrosis and the caspase-dependent cell apoptosis pathway^18^. Moreover, calpain activation has been observed in the *substantia nigra* of PD patients, and that inhibition of calpain activity attenuated neuronal degeneration and repaired behavioral functions in PD models^19^. In our research, calpain inhibitor Z-LLY-FM only partially rescued cell death, indicating that the 6-OHDA/AA not only activated the calcium-calpain pathway to induce cell apoptosis, but may also directly cause mitochondrial damage and caspase activation which lead to cells death.

Baicalein, a potent antioxidant and free radical scavenger, has been reported to exert a neuroprotective effect on ß-amyloid peptide-(25–35)-induced toxicity model, as well as on 6-OHDA-induced PD in both *in vivo* and *in vitro* models. The mechanism of neuroprotection of baicalein has been linked to its anti-inflammation, pro-differentiation and anti-apoptosis properties. Herein, we showed that baicalein exerts neuroprotective effects on 6-OHDA/AA-induced neurotoxicity. Through further investigation of the neuroprotective mechanism of baicalein, we found the specific mechanism of neuroprotection which involved the counteraction of intracellular calcium elevation, as well as secondary calpain activation and mitochondrial damage.

**Figure 4:**
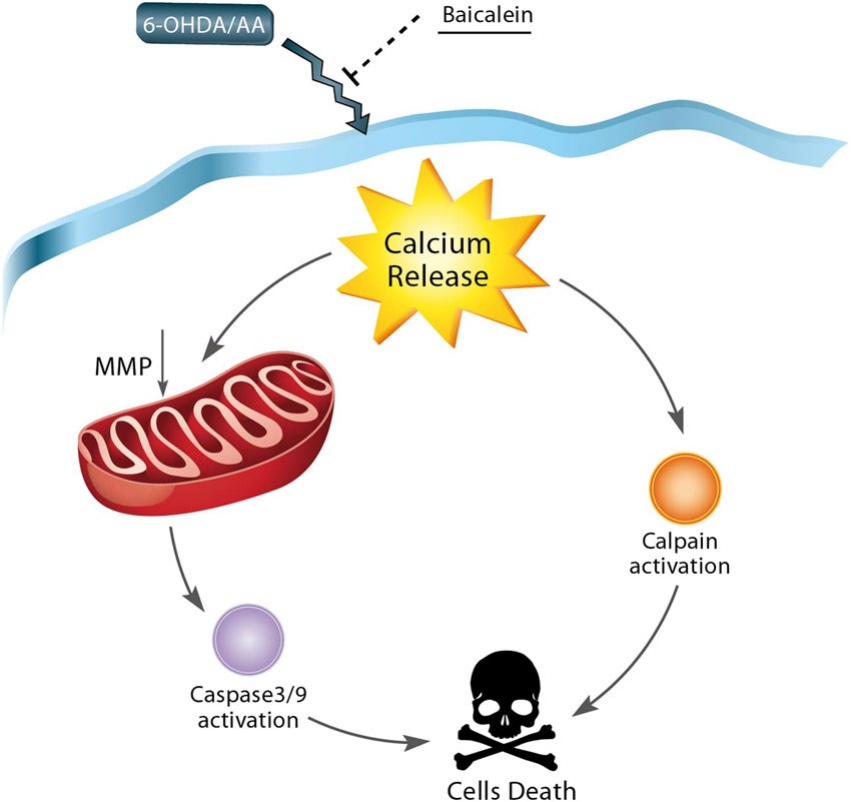
Proposed model for 6-OHDA/AA toxicity and the protective effect of baicalein. 6-OHDA/AA caused an elevation of intracellular calcium, which led to cell apoptosis via mitochondrial damage and calpain activation, during which baicalein protects SH-SY5Y cells against the disruption of intracellular calcium homeostasis and apoptosis induction.

In conclusion, our research confirms that AA enhances the toxicity of 6-OHDA in SH-SY5Y cells by a molecular mechanism mainly involving disruption of intracellular calcium homeostasis and apoptosis induction. The model for 6-OHDA/AA toxicity and the protective effect of baicalein are proposed in Fig. 4. Thus our study reveals the critic role of intracellular calcium in PD pathogenesis, and suggests the therapeutic potential of cell-permeable calcium chelators in the treatment of PD. The data further provides experimental evidence for the clinical use of ***Scutellaria baicalensis*** *-*containing decoction for the treatment of PD.

## Materials and Methods

### Chemical

6-OHDA, Ascorbic acid (AA), baicalein, Niferdipine, Verapamil and MTT were purchased from Sigma-Aldrich(St, Louis, Mo). DAPI (4’, 6-diamidino-2-phenylindole) and Calcium indicator Fluo-4-AM dye, BAPTA were purchased from Thermo Fisher Scientific (Waltham, MA). Calpain activity fluorometric assay kit was purchased from Biovision. Mitochondrial membrane potential detection kit (JC-1) was purchased from Beyotime biotechnology (China). Apoptosis antibody sample kit and ß-actin antibody were purchased from Cell Signaling Technology (Danvers, MA).

### Cell culture and treatment

SH-SY5Y cells were maintained at 37 °C in an incubator with 95% humidified atmosphere and 5% CO_2_ and were cultured in DMEM (Gibco, 12100–046), supplemented with 10% FBS (Gibco, 26140–079-a). SH-SY5Y cells were plated at a density of 2.0 × 10^5^ cells per well in 24-well plates overnight. Prior to drug treatment, the culture medium was replaced with 0.15% AA containing DMEM/F12. Then 6-OHDA (dissolved in 0.02% ascordic acid), in the presence or absence of baicalein and other reagents, was rapidly added into culture medium with gentle shaking. Cells were left in an incubator for 15 minutes. After incubation, the drug containing medium was replaced with normal culture medium, and cells were further incubated for different times.

### MTT assay

Cell viability was measured by MTT assay. Briefly, SH-SY5Y cells were plated at a density of 2.0 × 10^5^ cells per well in 24-well plates. After designed treatment and incubation for 24 h, the medium in each well was aspirated and replaced with fresh medium containing 1 mg/ml MTT. After 4 h of incubation at 37 °C, formazan crystals were solubilized in 250 ul DMSO. The absorbance was measured with microplate reader at 570 nm. Data are expressed as percentage of absorbance compared with the control group.

### Spectrophotometric determination of 6-OHDA autodegradation

The autodegradation of 6-OHDA was measured spectrophotometrically by monitoring the formation of colorful p-quinone at 490 nm, which was carried out in a cell-free system. Briefly, PBS alone and PBS containing 0.15% AA were maintained at 37 °C thermostatically. The autodegradation experiment was initiated by addition of 6-OHDA stock solution at a final concentration of 100 μM. Absorbance at 490 nm was monitored at preselected times (30 s, 1 min, 2 min, 5 min, 10 min, 15 min, 30 min) using a microplate reader.

### DAPI staining for assessment of cell apoptosis

SH-SY5Y cells were plated at a density of 2.0 × 10^5^ cells per well in 24-well plates overnight. After designed treatment and incubation for 6 h, the cells were washed with ice-cold PBS, stained with DAPI (500 nM in PBS) for 5 min at room temperature, and then were fixed for 15 min in 4% paraformaldehyde solution at room temperature. After rinsing the samples twice with ice-cold PBS, the fluorescent images were detected using an InCell 2000 system.

### Intracellular calcium measurement

Intracellular calcium level was measured using the calcium indicator Fluo-4. Briefly, SH-SY5Y cells were plated at a density of 2.0 × 10^5^ cells per well in 24-well plates or 8.0 × 10^5^ cell per 60 mm dish overnight. Before treatment, SH-SY5Y cells were loaded with Fluo-4-AM (1 μM) at 37 °C for 30 min, then washed with PBS twice and subsequently incubated for another 30 min to ensure Fluo-4-AM to be cleaved into Fluo-4 by intracellular ester enzymes. After designated treatment and incubation for 0.5 h, the cells were either examined under confocal microscope or were trypsinized and measured by flow cytometry using FACS cytometer (BD Biosciences). To quantify the fluorescence signal using the microplate fluorescence reader, SH-SY5Y cells were plated at a density of 5.0 × 10^3^ cells per well in 96-well plates, and then the cells received the same treatment as mentioned above. Finally the fluorescence intensity of the calcium was measured by microplate fluorescence reader and expressed as relative fluorescence unit (RFU) per cells.

### Calpain activity assay

SH-SY5Y cells were plated in 100 mm dishes overnight. The cells were treated with 6-OHDA/AA and incubated for 6 h, cells were suspended in extraction buffer and incubated on ice for 20 min, then centrifuged for 1 min and the supernatant was collected for protein concentration assay. Reaction buffer and calpain substrate were added to each tube, and tubes were incubated at 37 °C for 1 h in darkness. Samples were transferred to a 96-well black plate and read in a fluorometer equipped with a 400-nm excitation filter and 505-nm emission filter. The data were expressed as percentage of fluorescence intensity compared with the control group.

### Determination of Mitochondrial Membrane Potential (MMP)

SH-SY5Y cells were plated at a density of 2.0 × 10^5^ cells per well in 24-well plates overnight. 6 h after 6-OHDA/AA treatment in the presence or the absence of baicalein and incubation, the cells were washed with ice-cold PBS and incubated with 5 μM JC-1 in serum-free medium for 30 minutes at 37 °C in darkness. The samples were rinsed with PBS twice and were analyzed under a flow cytometry using FACS cytometer (BD Biosciences) for fluorescence intensity of monomeric form (green fluorescence) and aggregation (red fluorescence) of JC-1. The changes in MMP can be determined by comparing the ratio of red fluorescence and green fluorescence with the control group.

### Western blotting

SH-SY5Y cells were washed with ice-cold PBS and lysed with ice-cold RIPA buffer (150 mM sodium choride, 1.0% Triton X-100, 0.5% sodium deoxycholate, 0.1% sodium dodecyl sulfate, 50 mM Tris, PH 8.0), which supplemented with proteinase inhibitors and phosphatase inhibitors. Lysates were denatured in 1 x sample loading buffer and the equal amounts of protein (20 μg) were subjected to 15% SDS-PAGE, then the proteins were transferred to a polyvinylidene difluoride membrane. After blocking with 5% fat-free milk in Tris-buffered saline containing 0.1% Tween-20 (TBST) for 2 h at room temperature, the membranes were incubated with different primary antibodies overnight at 4 °C. The antibodies used in western blotting were β-actin (mouse, 1:2000), cleaved caspase 3, cleaved caspase 9, and cleaved PARP (mouse, 1:1000). The proteins were detected by chemiluminescence using an HRP substrate (GE Healthcare) after incubating the membrane with HRP-conjugated secondary antibody and washing with TBST. Secondary antibody was HRP-conjugated lgG anti-Rabbit (goat anti-Rabbit, 1:2000), used as needed. The images of blotting were quantified using ImageJ software (Wayne Rasband, NIH, Bethesda, MD).

### Statistical analysis

The significance of differences between two groups was assessed by non-parametric Mann-Whitney test, and the differences between multiple groups were assessed by non-parametric Kruskal–Wallis ANOVA, with Dunn’s as post-hoc multiple comparison tests. Calculations were performed with Prism software. The results are the mean ± SD values obtained in three independent experiments. Statistical significance was considered when *p < 0.05.
